# What vaccine inequity has taught us: a way forward through the lens of ideal and non-ideal theory

**DOI:** 10.1080/11287462.2025.2497602

**Published:** 2025-05-07

**Authors:** Florencia Luna, Felicitas Holzer

**Affiliations:** a Bioethics Program FLACSO Argentina and CONICET Argentina, Buenos Aires, Argentina; b Institute of Biomedical Ethics and History of Medicine, University of Zurich, Zurich, Switzerland

**Keywords:** Vaccine equity, COVAX, ideal and non-ideal theory, pandemic treaty, vaccine allocation

## Abstract

The equitable distribution of vaccines has emerged as a major issue in pandemic treaty negotiations following the COVID-19 pandemic. Failures in global procurement and distribution have been attributed to ineffective allocation mechanisms and a general lack of cooperation. More than four years after the onset of the pandemic, this article presents a perspective on how to achieve a more equitable global allocation of medical supplies for future pandemics, drawing on the distinction between “ideal” and “non-ideal” schemes of cooperation. We will consider two perspectives: first, improving solutions under current, non-ideal circumstances where non-cooperation dominates in the short and medium term, given the challenges of an uncooperative international landscape; and second, implementing long-term policies that aim at ideal proposals, assuming an increased level of cooperation in the future. This evaluation will address the past successes and shortcomings of the COVAX facility, and also the negotiations on a pandemic treaty led by the World Health Organizations, to better address future pandemics. We will discuss key issues that ought to be of central concern when moving towards more cooperative solutions in the future.

## Introduction

Global vaccine equity is a central tenet of global health. However, the final results of vaccine distribution during the COVID-19 pandemic were quite poor, given the post-pandemic vaccine roll-out, and the pandemic exacerbated global inequity (Emanuel & Persad, 2023).

To illustrate the scale of the vaccine equity problem, data from April 2021 showed that about 87 percent of all vaccines were administered in wealthier countries or regions when vaccine introduction began, while only 0.2 percent were allocated to low-income countries (Li et al., 2021). Vaccination rates in poorer countries were as low as 1 in 500 by 2021 (United Nations, 2021). Consequently, many poorer countries received significantly fewer vaccines in relation to excess deaths compared to high-income countries. Recent analysis also shows that low- and middle-income countries were inequitably treated in terms of vaccine distribution, meaning that many of them received inadequate vaccine allocations relative to their excess mortality (Emanuel et al., 2020; United Nations, 2021).

In this paper, we explore a way forward to achieve a more equitable global allocation of vaccines for future pandemics. This is particularly important in the light of the World Health Organization’s efforts to bring its member states together to resume negotiations after more than two years that failed to produce an agreement on the world's first pandemic treaty in May 2024. A follow up meeting in September did not change this outcome.^1^

So far, the COVID-19 pandemic has shown that cooperation mechanisms have led to sub-optimal vaccine equity outcomes. To address this, we propose to revisit the distinction between “ideal” and “non-ideal” policies. Policies that we call “ideal” require compliance with rules of cooperation between states and adherence to collectively fixed rules that can be enforced by the international community, while non-ideal policies do not. We are fully aware that complete cooperation and compliance is unlikely, as demonstrated by past international agreements and negotiation procedures (Wagner, 1983). However, the term “ideal” here refers to proposals that depend on the general commitment to adhere to (international) rules of cooperation and policies, even if sanctions might not be fully enforced. For us, both types of policy models can coexist and complement each other, with non-ideal solutions sometimes serving as satisfactory second-best options in the short and medium term.^2^

Our focus will be on evaluating both, already tested and proposed mechanisms to advance vaccine equity, with particular emphasis on the COVID-19 Vaccines Global Access Platform (COVAX) and other yet-to-be-realized policy proposals. Our analysis will encompass two perspectives: short- and medium-term policies that operate within the current, non-ideal circumstances, and long-term solutions that may envision a generally higher cooperation level of the international community. Past successes and failures in global vaccine distribution may serve as benchmarks to guide the ongoing negotiation of a pandemic treaty. To structure our analysis, we propose five normative criteria to assess both ideal and non-ideal policy proposals: political feasibility, effectiveness, permissibility, equity, and alignment with long-term policy goals.

## Challenges in the global vaccine distribution process

Policies implemented in the non-ideal context of the COVID-19 pandemic unveiled the current state in which cooperation is restrained by real-world conditions, such as partial compliance and no full cooperation. One of the significant challenges was the inefficiency and complexity of vaccine dose donations, which had been more difficult than initially anticipated. High-income countries hoarded large quantities of vaccines before sharing even the first doses with the rest of the world (Li et al., 2021; Valentini, 2012). Vaccine nationalism exacerbated this issue. By the end of 2021, nearly half of the world’s population had received two doses of a COVID-19 vaccine. But those vaccines were not distributed equitably, that is, vaccination rates were 75% in high-income countries, but less than 2% in some low-income countries (Ledford, 2022).

The establishment of COVAX was certainly a positive initial step towards promoting global vaccine distribution in a fair and equitable manner. Aside from PAHO’s Revolving Fund in the Americas, which is a regional allocation mechanism, COVAX marked the first mechanism for allocating medicines and vaccines at the *global* scale (Pan American Health Organization (PAHO), 2023). COVAX’s priority has been to provide vaccines to low-income countries, while allowing mostly high-income countries to purchase vaccines independently without restrictions on bilateral agreements (Gavi, 2024).

Yet, the COVAX facility faced a number of challenges during both the initialization and implementation phases. Overall, COVAX had delivered more than 1 billion COVID-19 vaccines to 144 participating countries by January 2022, against a target of 2 billion doses to low- and middle-income countries by the end of 2021 (Gavi, 2022). This shortfall was partly due to a chronic lack of funding (Holzer et al., 2023). In addition, the ethical basis for distribution was criticized, that is, the equity standard of proportional allocation, which does not take into account the urgency of health needs and excess mortality in individual countries (Emanuel et al., 2020).

Two competing standards of equity emerged: First, “proportional allocation” or “population-based allocation” proposed that vaccine supplies should be distributed equally among countries. COVAX, under the auspices of WHO, Gavi and CEPI, proposed a population-based or proportional allocation standard that would be sufficient to vaccinate 3% to 20% of the population initially.^3^ Second, a priority-based or health burden-based model (also known as the “fair priority model”, developed by scholars around Emanuel, Luna and colleagues (Emanuel et al., 2020; Emanuel & Persad, 2023) suggested that equity should be assessed on the basis of COVID-related health burden (e.g. measured by vaccines per excess death). The latter represents the current consensus among researchers (Emanuel et al., 2020; Emanuel & Persad, 2023).

Another problem has been that vaccine delivery has been hindered by the so-called “last mile” problem – logistical challenges in transporting vaccines from central distribution hubs to remote areas where persons with vulnerabilities live, often resulting in limited access to vaccines even after they have been delivered to low- and middle-income countries. Additionally, “last inch” problems emerged from a lack of trust in health systems and interventions by marginalized communities, vaccine hesitancy, and reduced vaccine uptake. The term “last inch” refers to challenges of ensuring that vaccines are actually administered to individuals (Lavery et al., 2023).

Also, a cluster of problems arose from failures in local vaccine procurement and distribution processes. Vaccine research, development, and manufacturing were disproportionately concentrated in the global North, with business models often neglecting the equity interests of public and non-profit investors, particularly in the global South. The international response to COVID-19 often conflicted with the profit-driven motives of pharmaceutical companies, which may have contributed to the prolongation of the pandemic, especially in Africa (Borges et al., 2022).

Furthermore, regional capacity building for vaccine production—one of COVAX’s goals—was either not addressed or insufficiently prioritized (Holzer et al., 2023). According to Li and colleagues, this was the case because COVAX lacked the enforcement power to compel governments or authorities to take collective binding actions (Borges et al., 2022; Li et al., 2021; Hotez, Bottazzi & Yadav, 2021). As a result, the initiative was primarily rhetorical, despite the involvement of WHO, CEPI, and Gavi.

The COVAX facility officially closed on December 31, 2023, after delivering nearly 2 billion vaccine doses to 146 countries and reportedly averting an estimated 2.7 million deaths after having fallen short in the most critical moments of timely and efficient deliveries (World Health Organization, 2023a). This result was made in the face of significant global challenges, including a lack of international cooperation, vaccine hoarding, geopolitical maneuvering, and the use of vaccines as a tool for soft power. As previously noted, the initiative’s main shortcomings stemmed from the fact that vaccines were not always prioritized for the regions that needed them the most.

## Analyzing past and future policy solutions

In the following, we will systematically examine past and potential future policy solutions for global vaccine allocation using a normative framework. Our goal is to provide insights that can help structure the discourse on short and long-term policy solutions regarding global vaccine equity. This analysis will be based on [Lunas, forthcoming]’s framework^4^ and will evaluate policies according to five criteria to identify the most effective normative solutions among various proposals under both ideal and non-ideal circumstances [Luna, forthcoming]. Specifically, we can assess the merits and shortcomings of (i) bilateral donations and (ii) the Global COVID-19 Vaccines Access Platform (COVAX). To this end, we propose a comprehensive framework for evaluating policy proposals, as outlined below:

C1: The proposal should be politically feasible.

C2: The proposal should be likely to be effective.

C3: The proposal should be morally permissible.

C4: Among competing proposals, there should be a preference for those that aim to address the most significant injustices.

C5: The proposal should align with long-term goals, such as virus control and pandemic preparedness, while avoiding obstacles to these goals.

Let’s look at the status quo – characterized by the lack of forced cooperation during the COVID-19 pandemic – through the lens of the five criteria in a rough initial assessment. Bilateral donations and purchase agreements were used by several countries to secure geopolitical advantages and exert soft power influence. The geopolitical dimension became particularly important as many countries sought to increase their influence through “vaccine diplomacy”. China, for example, was closely associated with this strategy, using vaccine exports to enhance its reputation and secure favorable geopolitical outcomes (Ledford, 2022; World Health Organization, 2023a). However, China was not alone in this practice; bilateral donations often surpassed multilateral initiatives in many countries (Ledford, 2022; World Health Organization, 2023a). It's also important to note that geopolitical considerations impacted COVAX as a multilateral cooperative [Holzer et al., 2023].

When analyzing bilateral donations, certain features can be identified at first glance: policies based on the criteria outlined can only be considered as truly meeting the first criterion, political feasibility (C1). Bilateral donations were ineffective (C2) in addressing the global impact of COVID-19, as many countries with high excess mortality rates were overlooked by donor countries, allowing the virus to continue circulating (Emanuel & Persad, 2023; World Health Organization, 2023a). Consequently, pressing equity issues (C4) were not adequately addressed. Additionally, the prioritization of donor countries’ interests did not align with moral permissibility (C3), when we put the criteria of saving lives, as significantly more lives could have been saved using alternative allocation mechanisms (Emanuel & Persad, 2023; World Health Organization, 2023a). Finally, bilateral donations were not aligned with long-term goals (C5) of effectively controlling virus spread and ending the pandemic equitably.

In contrast, and to give a brief first impression of the multilateral COVAX facility for global vaccine allocation, the facility appears to have been politically feasible (C1) and morally permissible, if not morally required (C3), in that it significantly promoted more equitable distribution and increased allocation of vaccines to poorer countries with significant health needs. Its aim was to prioritize vaccine donations to poorer countries and address significant injustices in the global access to medical supplied (C4). However, COVAX did not fully meet criterion C2 because it was not sufficiently effective in achieving its objectives and did not prioritize countries with the highest health burden, partly because of the equity standard of proportional allocation (De Bengy Puyvallée et al., 2022; Emanuel & Persad, 2023; World Health Organization, 2023a). Compliance with C5 was also challenging, as sustainable measures, particularly regional capacity building, were not adequately promoted despite their importance for pandemic preparedness [Luna, forthcoming]. Nevertheless, despite these challenges, COVAX performed better than bilateral donations regarding the criteria C1-C5.

## Solutions in the short- and middle-term regarding COVAX

Our framework also allows us to explore ways to improve COVAX. Despite its closure on 31 December 2023, COVAX has emerged as one of the most promising initiatives during the pandemic, offering concrete solutions for global vaccine distribution in the short and medium term, even if non-cooperation continues to dominate the international landscape for the foreseeable future. We argue that the founding organizations of COVAX – the World Health Organization, the Coalition for Epidemic Preparedness Innovations (CEPI) and the Vaccine Alliance Gavi among others – have scope to optimize their activities even in the absence of a binding pandemic treaty. We will therefore propose strategies that could improve the COVAX framework as it has been established in the past. Our recommendations aim to meet the criteria of political feasibility (C1) and effectiveness (C2), while offering a better solution for the remaining criteria (C3 to C5).

First, the allocation criterion of the COVAX facility could benefit from significant revision. Emanuel and colleagues (2020) argue persuasively that relying solely on population-based allocation – where the same standard is applied uniformly to all countries – does not take into account the different health burdens caused by the pandemic (Emanuel & Persad, 2023; World Health Organization, 2023a). Prioritizing countries with high excess mortality and greater health needs would significantly improve both equity and efficiency.

Second, as suggested earlier, it is essential that allocation mechanisms such as the COVAX facility, and in particular the World Health Organization (WHO), play a more proactive role in facilitating regional capacity building especially in middle-income countries where the necessary infrastructure is present. This includes improving infrastructure, training health workers and facilitating technology transfer between vaccine innovators and potential manufacturers. Key areas include improving access to reagents, investing in production facilities and supporting the development of local manufacturing capacity (World Health Organization, 2024).^5^ While India, as an emerging economy, has played an important role in vaccine production in the past, other promising middle-income countries with untapped production capacity could make a significant contribution. Strengthening the local production capacity in these countries, where some infrastructure already exists, should be a priority from the outset [blinded]. It should also be noted that India was the only middle-income country contracted to produce and distribute vaccines, but reached its limits when it was itself hit by a wave of COVID-19, demonstrating that reliance on a single middle-income country is a fragile undertaking and that capacity needs to be developed more systematically at the regional level.

Third, local access to vaccines remained a bottleneck. In this regard, COVAX could better benefit low- and middle-income countries if WHO focused on strengthening local health systems to improve “last mile” access to vaccines, particularly for marginalized and remote populations. As noted above, improving “last mile” access through government support and targeted vaccination campaigns is also critical to improving local access (see section 2.1) (Osterholm & Dall, 2020).

Fourth, the Coalition for Epidemic Preparedness Innovations (CEPI) has been instrumental in fostering partnerships between the public, private, philanthropic and civil society sectors in low- and middle-income countries. In the future, CEPI could strengthen its role by accelerating the development of vaccines specifically tailored to the needs of poorer countries and populations facing emerging infectious diseases.

Fifth, COVAX faced significant criticism for its transparency and accountability practices. Key concerns included the lack of publicly available contracts with vaccine manufacturers and minimal disclosure of vaccine prices. Human rights organizations such as Amnesty International pointed out that the opacity of COVAX’s agreements with pharmaceutical companies limited public scrutiny, especially given COVAX's heavy reliance on public funding from governments (Amnesty International, 2021). Furthermore, the lack of detailed audits or third-party verification of pricing and supply arrangements raised concerns that COVAX was unable to fully enforce manufacturers’ commitments to “minimum profit pricing”, potentially allowing companies to charge higher prices. Similarly, critics argued that stronger accountability mechanisms in COVAX would have improved equitable distribution, which is particularly important for low-income countries that rely heavily on COVAX for access to vaccines (Shackelford, 2021).

Implementation of these policy proposals would represent significant progress towards the long-term goals of pandemic preparedness and strengthening regional capacity, particularly in middle-income countries that have the infrastructure to effectively counter the negative impact of pandemics (C5). Yet, despite the great potential to improve facilities such as COVAX, this allocation mechanism alone may not be a sufficient solution in the long term and requires institutionalized cooperation, as follows.

## Current efforts to strengthen cooperation

The next step towards a deeper level of cooperation might be pandemic preparedness and international cooperation within the context of a pandemic treaty. Discussions on the pandemic treaty began in 2021 under the leadership of the International Negotiating Body (INB), established by the World Health Assembly (WHA), the decision-making body of the World Health Organization. However, as announced by the WHA in 2024, member states had temporarily failed to reach an agreement on a legally binding pandemic accord. Despite missing the deadline after more than two years of negotiations, WHO Director-General Tedros Adhanom Ghebreyesus remained optimistic that the 194 member states would eventually reach an agreement within the next six to twelve months (See Flechter, E. R. (2024), World Health Organization (2024)), eventually resulting in an agreement in mid-April 2025.

So far, negotiations over several drafts have raised many topics, some of which are particularly important for achieving improved global vaccine equity. Article 11 of the latest draft addresses arrangements for transferring technology to low- and middle-income countries (LMICs), while Article 12 proposes a system for countries to promptly share samples and genomic sequences of pathogens. In return, LMICs would receive pandemic-related products at affordable prices when a pandemic is declared (See Nature Editorial (2024), World Health Organization (2024)). Another important principle that is worth a deeper evaluation is the principle of “common but differentiated responsibilities” of LMICs and HICs for pandemic prevention, response, and health system recovery. The principle of “common but differentiated responsibilities” establishes the common governmental responsibility at a global level shared by nation states. The principle acknowledges that responsibility among countries is unequally distributed due to their different capacities. The principle has been previously in climate change and was formalized in the United Nations Framework Convention on Climate Change.^6^ Other fundamental topics include: (i) how patents and intellectual property for drugs and products needed to address the pandemic should be handled; (ii) access to medicinal products during a pandemic; and (iii) the roles and responsibilities of resource-rich states and corporations, such as pharmaceutical companies (Khosla et al., 2023).

Yet, over the past two years, many critical voices have highlighted several shortcomings and a weakening of the treaty. Burki (2023) raises concerns about the systematic lack of transparency in the negotiation process (Adebisi et al., 2023; Burki, 2023). Another issue is that provisions in the zero draft, which held the pharmaceutical industry accountable, have been weakened. Specifically, the requirements for pharmaceutical companies to improve access to medicines, provide transparent public reporting of drug prices, and ensure equitable data sharing and technology transfer for pandemic-related products have been diluted. Instead of imposing legally binding obligations on the industry, the latest draft of the Pandemic Accord appears to rely almost entirely on voluntary measures and “corporate social responsibility” (Khosla et al., 2023). Additionally, there is concern about the potential removal of the principle of common but differentiated responsibilities, as well as the “time-bound waivers of intellectual property rights” in ongoing negotiations (See Khosla et al., 2023, Emanuel and Persad (2023) and [blinded]).

## Moving towards cooperative solutions in the long run?

Charting a course towards an improved system of cooperation that adequately addresses equity concerns is still work in progress and is best viewed as a long-term goal. As many scholars in following a line of ideal theory of justice argue, real-world policy needs the model of “ideal” goals assuming full cooperation and compliance in order to know what kind of world order we envisage as we move from non-ideal to cooperative circumstances of justice (Valentini, 2012). So far, it is true that COVAX has been an immense effort to increase cooperation, and as outlined in the previous section, there may be relatively simple policy proposals to increase efficiency even in a non-cooperative environment. Yet, as far the current negotiations for a long-term cooperative scheme are concerned, an international agreement is needed and can complement an improved COVAX facility even though its current version remains far away from adequately addressing equity concerns, also in view of our criteria (C1) to (C5).

In addition to the policy proposals that are feasible in our current environment, where states are generally not fully cooperating, the most effective long-term goal of containing future pandemics will surely be achieved through enforcement mechanisms that prevent the exacerbation of national self-interest. These include the disproportionate hoarding of vaccines and the prohibition of unlimited bilateral deals and vaccine policies that mainly benefit donor countries and pharmaceutical companies. Promoting vaccine equity among countries should be the ultimate goal, although the challenge to full cooperation remains immense. In this last section, we will outline some of the pressing challenges that may impede equitable policies and fulfillment of our criteria, most notably effectiveness (C2), moral permissibility (C3), the attenuation of the most significant injustices (C4) and the alignment with long-term goals such as virus control and pandemic preparedness (C5). These concerns have also been addressed in several versions of the draft pandemic treaty, starting with the principle that HICs and LMICs have common but differentiated responsibilities to address the following issues, according to their respective capacities.

### Intellectual property

In this context, a much-discussed policy proposal during the COVID-19 pandemic relates to intellectual property laws and trade agreements governing vaccine production and procurement (Santos Rutschman & Barnes-Weise, 2021; Thambisetty et al., 2021). As many scholars have argued, current intellectual property laws and regulations on licensing and technology transfer do not adequately address the equity interests of public investment in vaccine research and production (Iacobucci, 2021). Trade agreements, in particular the World Trade Organization’s Trade-Related Aspects of Intellectual Property Rights (TRIPS) Agreement, have created barriers in this regard (Khosla et al., 2023). Still, in the April 2024 draft of the proposal for the WHO pandemic agreement, the added clause left little hope that intellectual property laws will change, regarding the following wording “intellectual property protection is important for the development of new medicines, recognizing concerns about its impact on prices and recalling that the Agreement on Trade-Related Aspects of Intellectual Property Rights (TRIPS Agreement) does not and should not prevent Member States from taking measures to protect public health (…)” (See WHO Nature Editorial (2024), p. 5). This vague formulation actually blurs the possibility of the international community to implement TRIPS. Under current WTO rules, the TRIPS Agreement actually allows member countries to propose waivers on intellectual property protections in cases of “exceptional circumstances”, which could include public health crises like a pandemic. During COVID-19, India and South Africa initially proposed a broad TRIPS waiver to allow more equitable access to vaccines and therapeutics, aiming to suspend patent rights temporarily to facilitate widespread manufacturing and distribution of essential medical products. However, this proposal encountered opposition from high-income countries like the U.K., Japan, and some EU nations, which argued that a waiver could discourage innovation and disrupt pharmaceutical supply chains (Mitchell et al., 2023). Eventually, in June 2022, the WTO adopted a partial waiver on COVID-19 vaccine, allowing limited use of protected data and vaccine patents after extensive negotiations. This decision significantly deviated from the broader waiver initially proposed by India and South Africa in 2020, which also included therapeutics and diagnostics. Critics argue that the current waiver is insufficient, as many developing countries still face barriers such as restricted access to trade secrets and limited manufacturing capacity, contributing to global vaccination inequities (See Gabriele, 2022).

### Transfer of technology and knowledge

A major shortcoming of the pandemic has been the lack of transfer of technology and know-how. Technology transfer in this context involves sharing manufacturing techniques and knowledge to empower more countries and companies, particularly LMICs, to produce vaccines locally. This process can be recognized as an important strategy to increase vaccine access in underserved regions, but is also related to significant challenges regarding intellectual property rights, production complexity, and infrastructure limitations (Borges et al., 2022).

One of the primary reasons technology transfer is critical for vaccine equity is the potential it creates for local production. Many LMICs had limited access to COVID-19 vaccines because they lacked the necessary manufacturing capacity. Through technology transfer, these regions could develop the capability to produce vaccines locally, which would accelerate distribution, reduce dependency on imports, and enhance regional health security^7^ Additionally, establishing local production capabilities would enable countries to bypass some of the bottlenecks in global supply chains, which often delayed the availability of essential vaccine components. This localized production could help stabilize supplies, as manufacturing processes would be less affected by global disruptions, such as those seen during the pandemic.

Technology and knowledge transfer is in part acknowledged by the current draft of the pandemic treaty, which discusses “transfer of technology and know-how for the production of pandemic-related health products” (Article 11), and “benefit-sharing” mechanisms (Article 12) and “equitable, timely and affordable access to pandemic-related health products” (Article 13) through a global supply chain network. To be effective, the knowledge transfer of technology should be required and steps for its implementation should be clear.

### The role of the pharmaceutical industry

One important other point that will directly impact on future cooperation is the attribution of responsibility to key private actors in addressing the pandemic, and most notably the pharmaceutical industry. Borges et al. (2022) critically examine the role of the pharmaceutical industry during the COVID-19 pandemic and highlight how profit-driven motives contributed to unequal access to vaccines globally. Specifically, their study shows how high-income countries received priority access and secured significant vaccine supplies through pre-purchase agreements, which boycotted the original idea of COVAX.

The authors further argue that the pandemic's challenges exposed weaknesses in global health governance, where the profit motives of pharmaceutical companies overshadowed equitable access goals. They recommend systemic changes to global health policies, such as an international treaty to enforce equitable access and a stronger regulatory framework to ensure that the pharmaceutical industry supports global public health rather than profit maximization in times of crisis (Borges et al., 2022, p. 3111f). In line with the previous section, a major proposal includes a TRIPS waiver to allow wider production capabilities in LMICs, although this is viable only if manufacturing capacity in these regions improves.

### Governance

A crucial step towards cooperation is the enforcement of principles and agreed rules. A pandemic agreement could indeed serve as a prerequisite for fostering cooperation and initiating a constructive discourse on setting more equitable standards for distribution and supporting manufacturing capacity and technology transfer. Yet, as suggested by Emanuel and colleagues, countries and their governments could have the option of opting for a limited number of bilateral agreements that would still allow them to vaccinate their own populations (Emanuel et al., 2021; [Holzer et al., 2023]). Here, an issue that needs to be considered in advance is the role of funding countries in the distribution of products. For example, countries collaborating with funds such as COVAX to develop and supply new vaccines should be given priority and an appropriate percentage of supplies, for example an amount sufficient to meet the most urgent health needs in a pandemic. In the COIVD-19 crisis, the risk of influenza was suggested (Emanuel et al., 2021; Gabriele, 2022). Such a policy will not only be fair, but will also encourage countries to cooperate and avoid the proliferation of bilateral agreements and increased vaccine nationalism (e.g. with a cap of 60% of doses retained by high-income countries).

Here, international bodies, led by the World Health Organization (WHO) and Gavi, could play a leading role in coordinating collaborative efforts and incentivizing cooperation between different stakeholders, including the pharmaceutical industry, NGOs, international organizations and nation states. These leading institutions should be supported by international public policies at the United Nations and the World Trade Organization, as well as by mechanisms and institutions established at national and regional levels to improve global health governance, such as the PAHO Revolving Fund (Pan American Health Organization (PAHO), 2023; Van de Pas et al., 2017).

It must be acknowledged that promoting such governance is an extremely ambitious and important endeavor, particularly in terms of feasibility (C1), even if our other criteria (C2 to C5) may be more easily met. Since the international arena is characterized by the absence of a global authority outside soft-law mechanisms, emerging global institutions often lack the ultimate legitimacy to prioritize the global good while deprioritizing the interests of their major funders. Therefore, the primary task of international actors and stakeholders is to enforce and sustain the democratization of global institutions and to hold them accountable, including through the process of negotiating a treaty that puts global equity at the center of the discussion – this is the only way to generate long-term legitimacy. For example, a future allocation mechanism following COVAX should ensure that countries have an equal say in the procurement and distribution of vaccines (Van de Pas et al., 2017).

## Conclusion

The experience of the COVID-19 pandemic showed that a lack of serious international cooperation led to vaccine inequity, excess deaths and morbidity, a delay in the resolution of the pandemic and exacerbated poverty in poorer countries. Bilateral donations alone have not been sufficient to achieve equitable vaccine distribution. It is essential that the international community develop long-term strategies that build on the initial efforts of COVAX – a useful first step in non-cooperative circumstances – while addressing past inefficiencies.

Higher levels of cooperation are urgently needed. While achieving full cooperation remains a challenge, also in light of the current pandemic treaty it is important to seek better policy solutions for vaccine equity in both the short and long term. In the short term, we propose that efficiency and alignment with pandemic preparedness and disease control objectives can be improved, even in the absence of full cooperation, such as through an enhanced COVAX facility to support the actions we propose. In the long term, the establishment of robust and binding international agreements addressing, at a minimum, the key issues we have discussed. This would certainly improve some of the current shortcomings and will be essential to provide a basis for cooperation at the global level. Given our past experiences, it is time to learn from them and commit more seriously to policy solutions that promote vaccine equity.

## Notes

1 See footnote 1: this article was written and accepted for publication before negotiations under led by the WHO on a pandemic treaty were concluded in mid-April 2025. World Health Organization (2024); Updates about the last hybrid meeting of the Intergovernmental Negotiating Body (INB) for a WHO instrument on pandemic prevention, preparedness and response from September 9 to 20 can be found at https://www.who.int/news-room/events/detail/2024/09/09/default-calendar/eleventh-meeting-of-the-intergovernmental-negotiating-body-(inb)-for-a-who-instrument-on-pandemic-prevention--preparedness-and-response; Nature Editorial (2024), Flechter (2024).

2 For further details regarding the distinction between ideal and non-ideal theory in Valentini (2012) and [blinded]. Valentini (2012) explains that ideal theory focuses on principles for a perfectly just society, assuming full compliance and ideal conditions, while non-ideal theory deals with justice under real-world conditions, considering partial compliance and practical constraints. Yet, ideal and non-ideal theories should be seen as part of a continuum, where ideal theory provides the ultimate goals of justice, and non-ideal theory offers practical ways to achieve incremental justice based on our imperfect reality. In our view, both are necessary for a complete understanding of justice.

3 (Emanuel et al., 2020) note 15.

4 See [blinded] for a full normative justification and explanation of the normative framework.

5 For reasons of completeness, it should be mentioned that this issue is currently under discussion in Article 10 of the draft version of the “Proposal for the WHO Pandemic Agreement” from April 22, 2024, as “tak[ing] measures, to provide support, and/or strengthen existing or newly created production facilities of relevant health products, at national and regional levels, particularly in developing countries, with a view to promoting the sustainability of such geographically diversified production facilities, including through supporting and/ or facilitating skills development, capacity-building and other initiatives for production facilities”

6 See Epstein, C. (2015). It is beyond the scope of this article to elaborate on this principle, but it is worth mentioning for the sake of completeness, and also to draw attention to an important discussion that raises pertinent questions about the equitable distribution of responsibilities in global health and pandemic preparedness.

7 [blinded] and Privor-Dumm et al., 2023.

## References

[CIT0001] Adebisi, Y. A., Aghogho Evaborhene, N., Olushola Ogunkola, I., & Oluebube Onyike, C. (2023). Beyond rhetoric in the pandemic treaty: Prioritizing equity and inclusion for marginalized populations. *Public Health Challenges*, *2*(3). 10.1002/puh2.115 PMC12039703 40496289

[CIT0002] Amnesty International. (2021). COVAX: Enhance transparency, share intellectual property. Article published on May 6, 2021, online available at https://www.amnesty.org/en/latest/news/2021/05/covax-enhance-transparency-share-intellectual-property-2/.

[CIT0003] Borges, L. C., Zeferino de Menezes, H., & Crosbie, E. (2022). More pain, more gain! The delivery of COVID-19 vaccines and the pharmaceutical industry’s role in Widening the Access Gap. *International Journal of Health Policy and Management*, *11*(12), 3101–3113. 36028975 10.34172/ijhpm.2022.6942 PMC10105197

[CIT0004] Burki, T. (2023). Calls for transparency in pandemic accord talks. *The Lancet*, *401*(10384), 1255. 10.1016/S0140-6736(23)00763-8 37062290

[CIT0005] De Bengy Puyvallée, A., & Storeng, K. T. (2022). COVAX, vaccine donations and the politics of global vaccine inequity. *Globalization and Health*, *18*(1), 1–14. 10.1186/s12992-022-00801-z 35248116 PMC8897760

[CIT0006] Emanuel, E. J., Fabre, C., Halliday, D., Leland, R. J., Buchanan, A., Tan, K. C., & Chan, S. Y. (2021). How many vaccine doses can nations ethically hoard? *Foreign Affairs*. March 9, 2021. Available at: https://www.foreignaffairs.com/articles/world/2021-03-09/how-many-vaccine-doses-can-https://www.foreignaffairs.com/articles/world/2021-03-09/how-many-vaccine-doses-can-nations-ethically-hoardnations-ethically-hoard.

[CIT0007] Emanuel, E. J., & Persad, G. (2023). The shared ethical framework to allocate scarce medical resources: A lesson from COVID-19. *The Lancet*, *401*(10391), 1892–1902. 10.1016/S0140-6736(23)00812-7 PMC10168660 37172603

[CIT0008] Emanuel, E. J., Persad, G., Kern, A., Buchanan, A., Fabre, C., Halliday, D., Heath, J., Herzog, L., Leland, R. J., Lemango, E. T., & Luna, F. (2020). An ethical framework for global vaccine allocation. *Science*, *369*(6509), 1309–1312. 10.1126/science.abe2803 32883884 PMC8691258

[CIT0009] Epstein, C. (2015). Common but differentiated responsibilities. *Encyclopedia Britannica*, Available at https://www.britannica.com/topic/common-but-dif-ferentiated-responsibilities.

[CIT0010] Farge, E., & Tétrault-Farber, G. (2024). WHO chief Tedros ‘confident’ of eventual pandemic treaty deal. Article published on May 27, 2024, online available at https://www.reuters.com/world/who-chief-tedros-confident-eventual-pandemic-treaty-deal-2024-05-27/.

[CIT0011] Flechter, E. R. (2024). Breaking – WHO Member States Fail to Reach Agreement on Pandemic Accord; Way Forward in Hands of World Health Assembly. Published on May 24, 2024, online available at https://healthpolicy-watch.news/breaking-pandemic-accord-negotiations-stall-again-with-way-forward-in-hands-of-world-health-assembly/.

[CIT0012] Gabriele. (2022). What happened to the COVID-19 vaccine patent waiver? *Harvard Law Bill of Health*, online available at https://blog.petrieflom.law.harvard.edu/2022/11/08/what-happened-to-the-covid-19-vaccine-patent-waiver/.

[CIT0013] Gavi. (2022). COVAX Deliveries. Available at https://www.gavi.org/covax-vaccine-roll-out.

[CIT0014] Gavi. (2024). What was the COVAX facility? Online available at https://www.gavi.org/covax-facility?gad_source=1&gclid=CjwKCAjw2dG1BhB4EiwA998cqH7Pv9RO-5fE1IpUdnSDgtFWx6N1wZd7QspTWGrEh4MHqwufJ_0DLxoC1YIQAvD_BwE.

[CIT1000] Holzer, F., Roa, T. M., Germani, F., Biller-Andorno, N., & Luna, F. (2023). Charity or empowerment? The role of COVAX for low and middle-income countries. *Developing World Bioethics*, *23*(1), 59–66. 10.1111/dewb.12349 35307947 PMC9111754

[CIT0015] Hotez, P., Bottazzi, M. E., & Yadav, P. (2021). Producing a vaccine requires more than a patent. *Foreign Affairs*, *10*, 1–4.

[CIT0016] Iacobucci, G. (2021). COVID-19: How will a waiver on vaccine patents affect global supply? *BMJ*, *373*, n1182. 10.1136/bmj.n1182 33972288

[CIT0017] Khosla, R., McCoy, D., & Marriot, A. (2023). Backsliding on human rights and equity in the Pandemic Accord. *The Lancet*, *401*(10393), 2019–2021. 10.1016/S0140-6736(23)01118-2 37271154

[CIT0018] Lavery, J. V., Porter, R. M., & Addiss, D. G. (2023). Cascading failures in COVID-19 vaccine equity. *Science*, *380*(6644), 460–462. 10.1126/science.add5912 37141365

[CIT0019] Ledford, H. (2022). COVID vaccine hoarding might have cost more than a million lives. *Nature News*, online accessible at https://www.nature.com/articles/d41586-022-03529-3. 10.1038/d41586-022-03529-3 36323903

[CIT0020] Li, Z., Lu, J., & Lv, J. (2021). The inefficient and unjust global distribution of COVID-19 vaccines: from a perspective of critical global justice. *INQUIRY: The Journal of Health Care Organization, Provision, and Financing*, *58*, 00469580211060992. 10.1177/00469580211060992 PMC8649905 34865544

[CIT1001] Luna (forthcoming). Lo ideal y no ideal en la bioética. Tomando decisiones en un mundo imperfecto. Eudeba (Buenos Aires), in press.

[CIT0021] Mitchell, A. D., Taubman, A., & Samlidis, T. (2022). The legal character and practical implementation of a TRIPS Waiver for COVID-19 vaccines. *Fordham Intellectual Property, Media & Entertainment Law Journal*, *33*(1), 100–145.

[CIT0022] Mitchell, A. D., Taubman, A., & Samlidis, T. (2023). Intellectual property and vaccine manufacturing: Utilizing existing TRIPS agreement flexibilities for COVID-19 and other public health crises. *Fordham Intellectual Property, Media & Entertainment Law Journal*, *25*, 1.

[CIT0023] Nature Editorial. (2024). A global pandemic treaty in sight: don't it scupper it. Published on May 21, 2024, online available at https://www.nature.com/articles/d41586-024-01464-z.

[CIT0024] Osterholm, M., & Dall, C. (2020). Episode 35: The Last Mile to the Last Inch: The Osterholm Update.

[CIT0025] Pan American Health Organization (PAHO). (2023). *PAHO Revolving Fund*. Available at: https://www.paho.org/en/resources/paho-revolving-fund.

[CIT0026] Privor-Dumm, L., Excler, J. L., Gilbert, S., Karim, S. S. A., Hotez, P. J., Thompson, D., & Kim, J. H. (2023). Vaccine access, equity and justice: COVID-19 vaccines and vaccination. *BMJ Global Health*, *8*(6), e011881. 10.1136/bmjgh-2023-011881 PMC10254992 37290896

[CIT0027] Santos Rutschman, A., & Barnes-Weise, J. (2021). The COVID-19 vaccine patent waiver: The wrong tool for the right goal. *Bill of Health*, *2021*, 1–5.

[CIT0028] Shackelford, E. (2021). Could COVAX be used as a test case to demand more aid accountability in South Sudan? *The New Humanitarian,* online available at https://www.thenewhumanitarian.org/opinion/2021/4/12/could-covax-be-test-case-to-demand-aid-accountability-in-south-sudan.

[CIT0029] Thambisetty, S., McMahon, A., McDonagh, L., Kang, H. Y., & Dutfield, G. (2021). The trips intellectual property Waiver proposal: creating the right incentives in patent law and politics to end the COVID-19 pandemic.

[CIT0030] United Nations. (2021). Unequal Vaccine Distribution Self-Defeating. World Health Organization Chief Tells Economic and Social Council’s Special Ministerial Meeting. Online available at https://press.un.org/en/2021/ecosoc7039.doc.htm.

[CIT0031] Valentini, L. (2012). Ideal vs. non-ideal theory: A conceptual map. *Philosophy compass*, *7*(9), 654–664. 10.1111/j.1747-9991.2012.00500.x

[CIT0032] Van de Pas, R., Hill, P. S., Hammonds, R., Ooms, G., Forman, L., Waris, A., Brolan, C. E., McKee, M., & Sridhar, D. (2017). Global health governance in the sustainable development goals: Is it grounded in the right to health? *Global Challenges*, *1*(1), 47–60. 10.1002/gch2.1022 28616255 PMC5445596

[CIT0033] Wagner, R. H. (1983). The theory of games and the problem of international cooperation. *American Political Science Review*, *77*(2), 330–346. 10.2307/1958919

[CIT0034] World Health Organization. (2023a). COVID-19 vaccinations shift to regular immunization as COVAX draws to a close. Online available at https://www.who.int/news/item/19-12-2023-covid-19-vaccinations-shift-to-regular-immunization-as-covax-draws-to-a-close.

[CIT0035] World Health Organization. (2024). WHO Member States agree to resume negotiations aimed at finalizing the world’s first pandemic agreement. Note published on March 28, 2024, online available at https://www.who.int/news/item/28-03-2024-who-member-states-agree-to-resume-negotiations-aimed-at-finalizing-the-world-s-first-pandemic-agreement.

